# Social validity of a contextual behavioral science-based intervention for retirement education

**DOI:** 10.1186/s41155-019-0137-0

**Published:** 2019-12-23

**Authors:** Leonardo Martins Barbosa, Sheila Giardini Murta

**Affiliations:** Department of Clinical Psychology, University of Brasília, Brasília, DF CEP 70910-900 Brazil

**Keywords:** Retirement, Aging, Social validity, Contextual behavioral science, Acceptance and commitment therapy

## Abstract

The literature shows that retirement can bring both positive and negative effects. However, there are few tested interventions for preparing workers for this transition and avoiding or minimizing its negative impacts. This paper presents a study with multiple groups that examined the social validity of an intervention for retirement education grounded in contextual behavioral science and acceptance and commitment therapy. Twenty-seven workers aged 29 to 65 divided into three intervention groups participated (group 1, *N* = 15; group 2, *N* = 9; group 3, *N* = 3). According to the participants’ evaluations, the intervention provided socially valid goals, socially acceptable procedures, and socially important effects. However, some improvements are still needed, such as the use of more dynamic methods, better formatted printed material, and increased fidelity between the content’s implementation and the prescribed activities. The positive results indicate that contextual behavioral science may bolster the development of interventions whose components possess evidence for their social validity. The further evaluation of the intervention via a clinical trial study will offer more robust evidence for its effectiveness. It is hoped that by increasing the availability of theory-based interventions in this area, the present study will promote valid strategies to facilitate better adjustment to retirement.

## Background

How does retirement impact a worker’s life? The answer is mixed: health can improve or worsen (van der Heide, van Rijn, Robroek, Burdorf, & Proper, 2013), as can satisfaction with life (Bonsang & Klein, 2012), marital life (Szinovacz & Davey, 2005), closeness to children (Szinovacz & Davey, 2001), leisure time (Nimrod, Janke, & Kleiber, 2008), and amount of physical activity (Kämpfen & Maurer, 2016), for example. Such variability stems from the personal and contextual resources available before and after retirement, which influence the impact of retirement and the worker’s adjustment to it (Wang, Henkens, & van Solinge, 2011). Thus, it is appropriate to invest in retirement preparation interventions that focus on maximizing the protective factors for adjustment to this phase of life.

Many of the factors which influence a worker’s retired life are known, notably: physical health, finances, psychological factors, leisure, the voluntariness of retirement, and social interaction (Barbosa, Monteiro, & Murta, 2016). However, few interventions have been developed and evaluated with the objective of modifying these factors (Boehs, Medina, Bardagi, Luna, & Silva, 2017; Hurtado & Topa, 2019; Pazzim & Marin, 2017). A review by Leandro-França, Murta, Hershey, and Barbosa (2016) consulted 13 databases in search of retirement education studies written in Portuguese, Spanish, or English that included the description and evaluation of the procedures and results. Encompassing publications before 2015, only 11 matched the inclusion criteria, all group interventions. Furthermore, many of the studies presented limitations in design, instrument quality, and/or a lack of robust data analysis. The scarcity of properly developed and tested interventions reduces the worker’s preparedness for retirement. In turn, this poor preparedness can increase the worker’s vulnerability to adjustment problems and reduce quality of life. To minimize such risks, developing and evaluating retirement education interventions more rigorously is recommendable.

### Intervention development and social validity

Intervention development is part of the research cycle of prevention and promotion in mental health (Murta & Santos, 2015). The cycle may be divided into seven stages (Muñoz, Mrazek, & Haggerty, 1996) related to the target problem: descriptive studies, to assess the problem prevalence and incidence; etiological studies, to identify the risk and protection factors for the problem in question; development studies, to construct and test the intervention’s viability and acceptability; efficacy and effectiveness studies, to evaluate the intervention’s effects; and diffusion and cultural adaptation studies, to transfer and adjust the intervention to other contexts and cultures. Intervention development is further divided into three substages (Rohrbach, 2014): theoretical foundation, which identifies the target-public, describes the problem and its theory-based determinants, and defines the objectives; program construction, which selects and integrates several techniques according to the context and the population’s characteristics; and the pilot, which evaluates the effects of the intervention’s individual activities and general outcomes. One of the expected products of this cycle is an intervention that participants evaluate as socially valid.

The social validity of an intervention encompasses the social significance of the goals, the social acceptability of the procedures, and the social importance of the intervention’s effects (Fawcett, 1991; Wolf, 1978). This concept arose at the end of the 1960s as a distinctive characteristic of applied behavior analysis, which differed from experimental behavior analysis by emphasizing the social relevance of the studied problems (Baer & Wolf, 1987). In the following years, the concept spread due to the increased community participation in interventions and currently it has at least 12 definitions (Carter, 2010).

Besides helping legitimize the results, social validity can also boost implementation quality (Lane & Beebe-Frankenberger, 2004), an aspect which deserves special attention. Durlak and DuPre’s (2008) review identified 23 factors that can influence the implementation quality of health prevention and promotion programs, organized into five categories: community factors, such as policies for preventing mental health problems and promoting good mental health; provider’s characteristics, such as the ability to implement the intervention; innovative characteristics, such as the possibility of adapting the intervention to the population; organizational capacity, such as institutional openness to change; and means of delivery, such as the training of the professionals who will conduct the intervention. Based on a sample of 542 studies, their results show that well-implemented interventions produce effects two- to threefold greater than less carefully implemented interventions. As the authors attest, implementation matters. However, none of the interventions reviewed by Leandro-França et al. (2016) include evaluations of social validity.

### A systematic approach for the development of interventions

Six decades ago, Kurt Lewin remarked that “nothing is as practical as a good theory” (Lewin, 1951), p. 169. A good theory helps to identify the determinants of behavior and the process of change, besides guiding the choice of intervention techniques, maximizing the results, or pointing out necessary changes (Bartholomew & Mullen, 2011). On the other hand, an implicit and inarticulate theoretical foundation muddles the understanding of the workings and adaptation of an intervention (Herbert, Gaudiano, & Forman, 2013). Such problems may have compromised the consistency of the majority of retirement education interventions that are not explicitly theoretically based (Leandro-França et al., 2016): out of the 11 articles reviewed by the authors, only five mention the theories that guided the intervention development—FRAMES model (F = feedback, R = responsibility, A = advice, M = menu of options, E = empathy, S = self-efficacy), transtheoretical change model, social cognitive theory, appropriate retirement decision model, and image theory. As such, retirement education studies may lack strong conceptual bases.

One proposal to overcome this issue is to guide the development of the various knowledge construction stages by common principles, emphasizing a natural cohesion among the different levels and dimensions of knowledge. Contextual behavioral science (CBS) (Vilardaga, Hayes, Levin, & Muto, 2009) fits this objective. CBS is a science development strategy that integrates applied basic principles, theories, and models that attempt to predict and influence the actions of individuals and groups. Its research program includes philosophical assumptions, basic and applied theory, and intervention development. In essence, it consists of a systematic application of contextual thought to different levels and dimensions (Pepper, 1942).

CBS is based on functional contextualism, a pragmatic system characterized by four essential criteria (Biglan & Hayes, 1996; Hayes, Hayes, & Reese, 1988). The first is the prediction-and-influence of behavior: prediction and influence as a single objective in the sense that only analyses that include both are considered valid (Hayes et al., 1988). Second is the act-in-context as the basic unit of analysis: the necessity of considering an event in the historical and environmental situation in which it occurred. Third comes functionality as a criterion for truth; regardless of the considerations of a possible underlying structure of the world, the analyses useful for the previously established objectives are considered true. In an analysis, variables are sought that permit predicting an event’s occurrence as well as influencing its likelihood in the groups where that event can be modified. In fourth place comes a unitary vision of the human being, understood as a unified organism. Thus, thoughts, feelings, and behaviors are considered actions on the same level, without hierarchy (Hayes & Brownstein, 1986).

CBS extends its pragmatism to several theories, understood as systematic and comprehensive analysis of acts-in-context with the purpose of predicting-and-influencing the actions of individuals and groups (Hayes, Barnes-Holmes, & Wilson, 2012). Relational Frame Theory (Hayes, Barnes-Holmes, & Roche, 2001) is an example of a basic theory with practical applications. It is a theory of cognition and human language based on the learned ability to relate symbols arbitrarily. Understanding the function and context of those relationships may allow predicting and manipulating their subjective impact.

At the level of intervention development, acceptance and commitment therapy (ACT) is an example of approach based on the contextual principles (Barbosa & Murta, 2014; Hayes, Strosahl, & Wilson, 2012). ACT is a clinical model of health, psychopathology, and intervention that aims to promote psychological flexibility: the ability to maintain or change behavior, in a conscious and intentional manner, as a function of one’s values (Hayes, Luoma, Bond, Masuda, & Lillis, 2006). While the flexibility comes from the functional use of psychological mechanisms, the dysfunctional use of the same mechanisms can result in psychopathology. Finally, CBS is a comprehensive strategy for the promotion of science. It is based on fundamental principles which are subject to updating and it seeks to integrate areas of knowledge that share common principles, thereby improving the development of knowledge itself. Besides integrating different dimensions and levels of knowledge, CBS also indicates criteria to guide the scientific research agenda, as seen in various articles (Hayes, Long, Levin, & Follette, 2013; Hayes, Strosahl, & Wilson, 2012; Vilardaga et al., 2009). For example, a study by Hayes et al. (2013) suggests several ways to improve the intervention development process, such as coordinating research concerning interventions and philosophical assumptions; emphasizing functional processes, which must be manipulable; planning and disseminating the implementation after the initial stages; adopting models applicable to the public health care dimension; precocious effectiveness testing; and researching transdiagnostic comprehensiveness.

In short, retirement education intervention development seems two have two significant gaps: interventions that lack explicit theoretical foundation and a scarcity of resources. In this context, the construction and social validation of a retirement education intervention and the application of CBS as a guide for this process is a first step in closing both of these and complementing the current literature. Hence, the present article’s objective is to describe the development and examine the social validity of a retirement preparation intervention based on CBS. Specifically, the study aims to examine the social significance of its goals, the social acceptability of its procedures, and the social importance of its effects.

## Method

### Participants

Given the intervention’s stage of development, a multiple group study was done. Retirement education applied to three groups of workers transitioning to retirement from three different contexts in the city of Brasilia, Brazil, between August 2015 and December 2015 was analyzed. According to the original schedule, the groups’ interventions were to be done sequentially so problems identified in one group could inform changes to the subsequent one(s). However, due to the institutions’ availabilities, the schedule was changed and there was some overlap in the implementations.

Altogether, 27 workers aged 29 to 65 (*M* = 61) participated in the study (22 women and five men). Group 1 had 15 workers, 13 women and 2 men; group 2, nine, seven women and two men; and group 3, three participants, two women and one man.

Group 1 took place in a federal institution which manages science policies; group 2, in an arm of the federal justice department; and group 3, with workers identified through a professional researchers’ network. The only inclusion criterion was interest in participating in the intervention, regardless of age or the amount of time until or after retirement.

### Instruments

*Needs Assessment and Results Evaluation Questionnaire*. This information was collected through open questions developed by this study’s authors. At the beginning of the intervention, participants stated their needs and their expectations regarding the activity respectively by completing the sentences, “I sought out this activity because …” and “By the end of this activity, I expect to....” To indicate the achieved results, at the end of the intervention, participants completed the sentence, “When I compare my expectations with my results, I think that ….”

*Valued Living Questionnaire* (VLQ) (Wilson, Sandoz, Kitchens, & Roberts, 2010). The themes to be worked on each encounter were identified and selected using a version of the VLQ translated into Portuguese by the present study’s authors. Through a 10-point Likert-type scale, the instrument assesses the personal relevance of ten life domains and how much the individual’s behavior is oriented toward them.

*ACT Matrix* (Polk & Schoendorff, 2014). This is a diagram based on the ACT model that undertakes to promote psychological flexibility. It helps the individual or the group to discriminate (1) mental/sensory stimuli from behaviors and (2) living congruently from living incongruently with one’s own values. The matrix consists of the intersection of two perpendicular lines: a horizontal line that separates mental/sensory stimuli (lower half) from externally observable behaviors (upper half) and a vertical line that separates what is incongruent with values (left side) from what is congruent with values (right side). This cross-like diagram creates quadrants into which experiences are categorized: aversive internal events (lower left), dysfunctional actions (upper left), values (lower right), and goals (upper right). This categorization allows participants to identify their own themes (life domains) and values of interest as well as the behaviors they are willing to commit to.

*Satisfaction Perception Scale*. A scale developed by the present study’s authors to assess satisfaction with the intervention. On a 10-point Likert-type scale, at the end of each meeting, the participants expressed their satisfaction with the facilitator, content, material, and the intervention in general. Additionally, they could describe the positive and negative aspects of each item.

*Behavioral Activation Table* (Kanter, Busch, & Rusch, 2009). This instrument is used to promote the practice of new behaviors. The table comprises four columns in which the participant describes a behavior, the contingencies for its fulfillment, its potential obstacles, and the possible solutions for such obstacles.

*Activity Monitoring Chart* (Kanter et al., 2009). This instrument attempts to identify behavioral patterns and their frequencies. It consists of a table with two columns. The first lists ten life domains (for example, health or social life) and the second provides space for the participant to record the daily activities undertaken in each area.

### Procedures

#### Intervention

The intervention will be described according to the Template for Intervention Description and Replication Checklist and Guide (TIDieR) (Hoffmann et al., 2014):

*Brief name*: The intervention was not named.

*Why*: The intervention was developed based on retirement education interventions described in the literature (Leandro-França et al., 2016; Murta et al., 2014). It is theoretically based on Contextual Behavior Science and on Acceptance and Commitment Therapy. CBS is a science development strategy focused on principles that promote integration between applied basic principles, theories, and models that attempt to predict and influence the actions of individuals and groups. Its research program includes philosophical assumptions, basic and applied theory, and intervention development. ACT is a clinical model of health, psychopathology, and intervention that aims to promote the ability to maintain or change behavior, in a conscious and intentional manner as a function of one’s values.

*What* (*materials*): Printed materials were used, including a list of values and a list of retirement adjustment predictors. The list of values aggregates expressions obtained from the Survey of Guiding Principles (SGP) (Ciarrochi & Bailey, 2008). The list presents expressions that participants can choose or adapt to identify their own values, such as connection with nature, honesty, helping others, loyalty, romantic relationships, practicing sports, and eating healthy. The list of predictors was based on an integrative literature review about retirement adjustment predictors (Barbosa et al., 2016). Only predictors with broad empirical bases and evidence for positive effect were presented and discussed: psychological and physical health, finances, leisure, voluntary retirement, social interaction, retirement preparedness, marital relationship, post-retirement work, work conditions before retirement, spirituality, retirement length, parenting, and education.

*What* (*procedures*): Table 1 summarizes activities developed in each meeting for the three groups*.* Procedures included informative activities, such as presentation of retirement adjustment predictors, and practical activities, such as discussions about the themes chosen by the participants, values, and averse internal events. The number of life domains addressed in each group, however, varied according to the number of meetings: in group 1, two domains of life were addressed; in group 2, three domains; and in group 3, five domains.

**Table 1:** Content of the meetings

Meeting	Activity	Group
1	Presentation of the intervention	1, 2, 3
	Predictors of adjustment to retirement	1, 2, 3
	Presentation of the ACT Matrix	1, 2, 3
	List of values	1, 2, 3
	Task: activity monitoring chart	1, 2, 3
	Meeting evaluation	1, 2, 3
2	Results of previous meeting evaluation	1, 2, 3
	VLQ: three main life domains	1, 2, 3
	ACT Matrix: first life domain	1, 2, 3
	Task: behavioral activation table	1, 2, 3
	Task: describing first life domain relevance	1, 2, 3
	Meeting evaluation	1, 2, 3
3	Results of previous meeting evaluation	1, 2, 3
	Task review	1, 2, 3
	ACT Matrix—second life domain	1, 2, 3
	Task: behavioral activation table	1, 2, 3
	Task: describing second life domain relevance	1, 2, 3
	Meeting evaluation	1, 2, 3
4	Results of previous meeting evaluation	2, 3
	Task review	2, 3
	ACT Matrix—third life domain	2, 3
	Task: behavioral activation table	2, 3
	Task: describing third life domain relevance	2, 3
	Meeting evaluation	2, 3
5	Results of previous meeting evaluation	3
	Task review	3
	ACT Matrix—fourth life domain	3
	Task: behavioral activation table	3
	Task: describing fourth life domain relevance	3
	Meeting evaluation	3
6	Results of previous meeting evaluation	3
	Task review	3
	ACT Matrix—fifth life domain	3
	Task: behavioral activation table	3
	Task: describing fifth life domain relevance	3
	Task: individual plans for retirement	3
	Meeting evaluation	3
7	Results of previous meeting evaluation	1, 2, 3
	Task review	1, 2, 3
	Next steps	1, 2, 3
	Intervention evaluation	1, 2, 3

*Who provided*: The intervention was facilitated by the first author, who had 9 years of experience working as a clinical psychologist and 5 years as a group facilitator at the time of the study.

*How*: The intervention was delivered face-to-face in group format.

*Where*: Groups 1 and 2 met in institutions and group 3, in a private office. The three settings were similar: a private room with individual desks, windows, air-conditioning, and video projectors and screen.

*When and how much*: In group 1, four monthly 3-h meetings were conducted in an open group (accepting new participants throughout the meetings), totaling 12 h. In group 2, five weekly 2-h meetings were held in a closed group (new participants would not be admitted after the first meeting), totaling 10 h. In group 3, seven 90-min weekly sessions were conducted in a closed group, totaling 10.5 h.

*Tailoring*: The intervention focused on social validity and the authors expected to adapt it as much as possible according to participant feedback while in progress. To reach that goal, every meeting was concluded with an evaluation. The feedback was then evaluated by the authors, who implemented the changes in the following meeting. When this was not possible, changes affected the development of the following groups. Table 1 summarizes how changes were implemented throughout the groups.

*Modifications*: Initial design and dosage proposed closed groups, with four weekly sessions, each session lasting 3 h, totaling 12 h. However, in group 1, the institution coordinator requested the authors to allow new participants and offer monthly sessions. Both suggestions impacted the intervention negatively, so groups 2 and 3 were closed and had weekly sessions. In group 2, participants preferred to reduce each meeting duration (from 3 h to 2 h) and to increase the number of meetings (from four to five). In group 3, the number of meetings was increased again because group 2 participants had said they would like to work additional life domains. In order to minimize impacts in dosage, meeting duration was reduced to 90 min.

*How well* (*planned*): Intervention fidelity was not evaluated.

*How well* (*actual*): Intervention fidelity was not evaluated.

#### Data collection

Participants individually answered the Needs Assessment and Results Evaluation Questionnaire and Valued Living Questionnaire in the first meeting. The Activity Monitoring Chart was assigned in meeting 1 as homework to be answered daily by each participant until meeting 2. The ACT Matrix was used in meetings 2 through 7, inclusive. Participants answered it collaboratively in group. The Behavioral Activation Table was answered individually each meeting after answering the ACT Matrix. The Satisfaction Perception Scale was applied at the end of each meeting and answered individually.

#### Data analysis

The data analysis considered the distinctiveness of each group, the variability of contexts and groups, and their relative contribution to understanding the evidence for social validity of the goals, procedures, and effects. Hence, each group was analyzed relative to one of the three social validity components. Textual data were analyzed separately by two judges according to the procedure suggested by Braun and Clarke (2006). First, the participants’ answers were read over several times and then divided in basic units of analysis, each containing only one characteristic, positive or negative. Each unit was classified according to the theme and the themes were organized into more comprehensive categories. These categories were then compared to the original data and the discrepancies led to refinement of the final thematic categories. The quantitative data were tallied and analyzed by means of two descriptive statistics, frequency and mean.

In group 1, it was analyzed the social significance of the goals, based on the use of the ACT Matrix. This analysis was based on five criteria proposed by Lane and Beebe-Frankenberger (2004): the preferences and values of the consumer, the promotion of common skills between peers that are relevant in different contexts, the option to choose goals of personal interest, and the option to take advantage of pre-existing skills or to adapt aspects of the treatment. The participants’ reports were organized accordingly, indicating whether a given criterion had been met. In group 2, it was analyzed the social acceptability of procedures through reports and satisfaction scores. The satisfaction reports obtained for each item (facilitator, material, content, and general) were interpreted by identifying the aforementioned themes and their frequency. The satisfaction scores obtained for each item were grouped by meeting and presented as mean values for each category. In group 3, it was analyzed the social importance of the effects via the commitment to their goals and the final evaluation of the results. The commitment was evaluated through a weekly review of the goals, comparing a participant’s performance to the goal established in the prior meeting. The final evaluation of the results was analyzed in contrast with the reported initial expectations, revealing whether the intervention met the expectations.

## Results

### Group 1

**Fig. 1:**
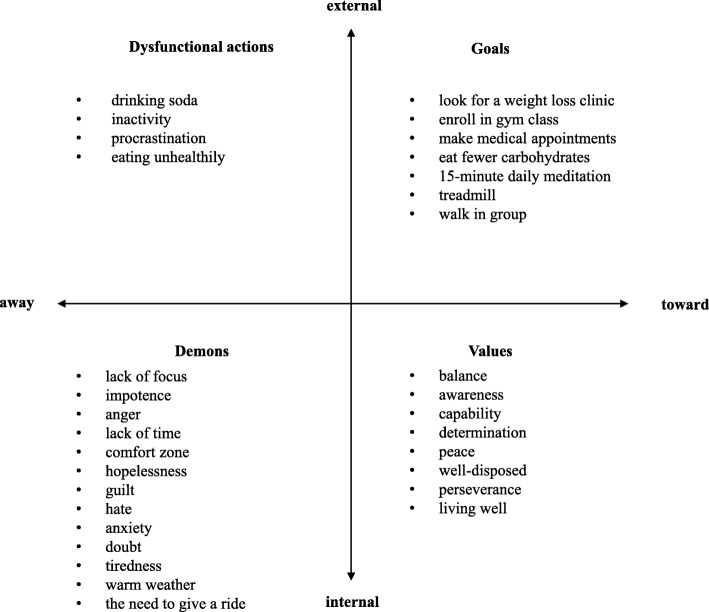
Matrix built by the group on the theme of health

Group 1 was the first intervention implementation and it remained open to new participants. A total of 15 individuals participated, with around nine present at each meeting. Data from this group was used to analyze the social significance of the themes. To this end, the results of two activities done in the third meeting will be presented: the task review and the filling in of the matrix. Figure 1 present the matrix filled in meetings 2 and 3 regarding the theme of health.

**Fig. 2:**
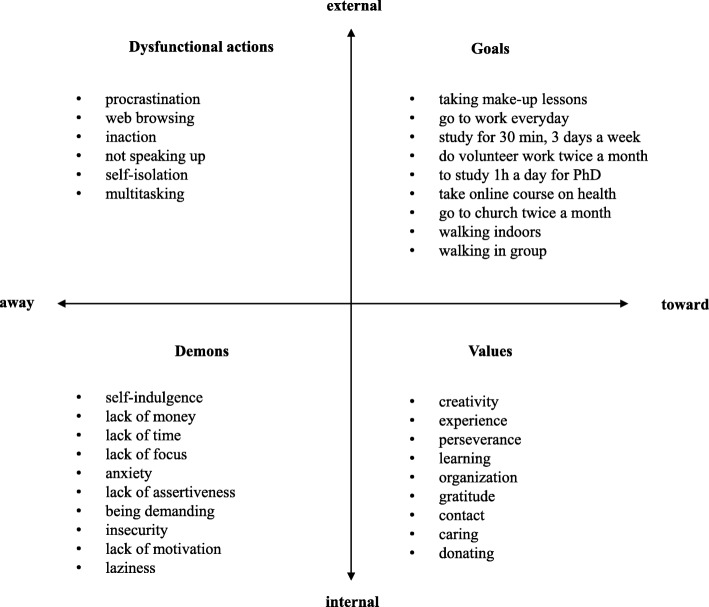
Matrix built by the group on life areas

Figure 2 presents the matrix as filled out in meeting 3. This time, the participants chose different life areas: career, education, citizenship, and spirituality. The possibility of addressing different areas concomitantly reveals two important characteristics of the instrument employed. On the on hand, it allows individualization of one’s own retirement preparation—values, obstacles, and goals according to personal interests. On the other, it promotes collaboration among the participants who share objectives and difficulties even when working on different themes.

In goal definition, the participants also worked together: for example, the task of “walking in a closed environment” was agreed upon by four participants who planned to do the activity together at lunchtime in the institution’s courtyard. The “walking in group” activity was agreed to by two participants who live near each other and planned to exercise before work.

### Group 2

Group 2 was the second implementation of the intervention. Based on the experience with the previous group, this group was closed. Two of the nine participants dropped out over the course of the meetings. At the request of the agency’s human resources coordinator, the intervention was increased to five meetings.

Data from this group was used to analyze the social acceptability of the procedures. To this end, the results of the qualitative and quantitative satisfaction evaluations done at the end of the meetings will be presented. Table 1 shows the positive and negative aspect categories reported by the participants, as well as the average satisfaction with each intervention item.

There were 37 positive comments about the facilitator, classified into four categories: assertiveness (*n* = 13, e.g., “objective”), flexibility (*n* = 13, e.g., “willing to listen”), attentive to the present (*n* = 8, e.g., “connected with the group”), and satisfying (*n* = 3, e.g., “I liked it”). There were eight negative comments, classified into three categories: conduct (*n* = 4, e.g., “I’m finding the pace fast”), time (*n* = 2, e.g., “too little time”), and clarity (*n* = 2, e.g., “sometimes I don’t understand where the activity is going”).

There were 19 positive comments regarding the content and procedures that were classified into four categories: productive (*n* = 8, e.g., “allowed for introspection”), clarity (*n* = 6, e.g., “enhanced the program’s objective”), satisfaction (*n* = 3, e.g., “appropriate”), well-tailored (*n* = 2; “focused on the group’s rhythm”).

On the negative side, there were nine comments grouped into three categories: poor instruction (*n* = 4, e.g., “have more practical examples”), lack of time (*n* = 4; “was hampered by too little time”), and low social interaction (*n* = 1; “absent coworkers could have added a lot”).

The material garnered 19 positive comments classified into three categories: satisfaction (*n* = 10, e.g., “I liked it”), instruction (*n* = 7, e.g., “instructive”), and responsiveness (*n* = 2; “my previous complaint was addressed”). There were seven negative comments, which were grouped into two categories: improving the material (*n* = 5, e.g., “the lack of numbering hinders using the material during the meeting”) and incompleteness (*n* = 2, e.g., “I wanted to see more than what the facilitator brought”).

As for the general evaluation, there were 17 positive comments classified into three categories: satisfaction (*n* = 7, e.g., “very good”), personally beneficial (*n* = 4; “I was happy for having listed suggestions and responded”), and social interaction (*n* = 2; “great group work”). On the other side, there were nine negative comments grouped into three categories: lack of social interaction (*n* = 3, e.g., “lots of people absent”), general procedures (*n* = 3, e.g., “balance the lecture part”), and personal problems (*n* = 3, e.g., “at this point in my life, I’m focused on my ghosts.”).

In total, there were 121 comments, 88 positive and 33 negative. The matrix was another important element. Although it was not cited in the comments, adhesion on the part of the participants qualified both to identifying values, objectives, and obstacles and planning changes.

Three other procedures also had undesired consequences. First, in group 1, the intervention was open to new participants after the first meeting. As such, it was necessary to re-present it several times, which undermined group consolidation and the continuity of work. To avoid this problem, new participants were not allowed to join the following interventions after the first meeting. Second, in groups 1 and 2, the intervention happened at the participants’ workplace and professional demands hampered their adherence. On the other hand, offering the intervention in the institution itself made it more accessible to the participants. Third, in group 2, the institution’s coordinator announced the intervention only to colleagues who had participated in other life quality programs, including a long-term retirement preparation intervention.

### Group 3

Group 3 was the last implementation and had three participants selected by convenience. To promote the addressing of more life areas, two additionally meetings were added, making a total of seven. To avoid large difference in workload, the meeting duration was reduced to 1.5 h. Thus, while group 2 had a workload of 10 h, with five 2-h meetings, the workload of group 3 was 10.5 h from seven 1.5-h meetings.

Data from this group was used to evaluate the social importance of the effects. For this, the behavioral effects resulting from the meetings will be presented. Table 2 shows the individual routines in the first week of the intervention. It can be seen that physical exercise is quite common, with a large variety of activities. Although participant 1 is retired from a career in medicine, she regularly undertakes activities in her post-retirement career as a teacher. Observe as well as participant 3 did not take part in activities with friends and that all her leisure activities are solitary ones. Table 2 covers only the areas all participants worked in the intervention; so the fields of “friendship” (participants 1 and 2) and “intimate relationship” (participant 3) were not considered. In Table 3, only activities related to the life areas worked during the intervention are presented.

**Table 2:** Qualitative and quantitative evaluations of meeting satisfaction

	Positive aspects		Negative aspects		Average satisfaction
	Category	*n*	Category	*n*	
Facilitator	Assertiveness	13	Direction	4	9.2
	Flexibility	13	Clarity	2	
	Attention to the present	8	Time	2	
	Satisfactory	3			
Content and procedures	Productive	8	Instruction	4	9
	Clarity	6	Time	4	
	Satisfactory	3	Social interaction	1	
	Tailored	2			
Material	Satisfactory	10	Material	5	9
	Didactic	7	Integrity	2	
	Responsive	2			
General	Satisfactory	8	Social insertion	3	9.2
	Personal benefit	5	Procedures	3	
	Social interaction	4	Personal matters	3

**Table 3:** Type and frequency of activities during the first week

Participant 1	*F*	Participant 2	*F*	Participant 3	*F*
Health					
Physical therapy	5	Exercise	6	Exercise Physical therapy	1
Exercise	6	Medications	7		3
Meditation	6				
Healthy eating	1				
Career					
Yoga lessons	2	Regular activities	6	Regular activities	5
Yoga studies	3				
Mantras course	1				
Leisure					
Breakfast in the bakery	1	Radio in the car	2	TV	5
Reading	4	Conversation with son	1	Shopping	2
Music	1	Bike ride with son	1	Lunch	1
Swimming pool	2	Supermarket	1	Dinner	1
Sauna	1	TV	1	Travel	1
Newspaper	2	Visit to relatives	1		
Family afternoon	1	Son’s basketball match	1		
TV	2				
Gongo	1				
Cinema	1				
Lunch with relatives	1				
Gardening	1				
Friendship					
N/A		N/A		–	
Close relationships					
Breakfast	1	Dialog during meals	5	N/A	
Lunch (2)	2	Son’s basketball match	1		
Exercise	1	Organizing living room	1		
Shopping	1	Visit to relatives	1		
Afternoon with daughters	1				
Dialog during meals	1				

*F* is frequency measured in days

Tables 4, 5, and 6 present individual data on the expectations, new behaviors, and the evaluation of intervention effects. The expectations section contains the motive for and expectations of participating in the intervention, which were collected at the beginning of the first meeting. The goals section lists each meeting’s theme and the monitoring of respective goals, done starting from the second week. The final evaluation section, answered in the last meeting, has the participants’ evaluations of the intervention’s effects.

**Table 4:** Expectations, goals, and evaluations of participant 1

Expectations					
Question	Participant’s answer				
Why did I seek out this activity?	I need more focus, more reality, to be more “grounded,” and to get better organized regarding time; opportunities to talk, work, organize, and put into action what I want to do from now on; to talk and deal with the fear of the new and the unknown path.				
What do I expect to achieve by the end of the activity?	To have put more order in and higher priority on what I want to do; to have guidance for how am I going to do what I want to do; to have gained some “capacity” or tools to deal with my insecurity and fear – so these do not hinder me.				
Goals					
Activity	Life domain and meeting number				
	Health meeting 2	Career meeting 3	Leisure meeting 4	Career meeting 5	Couples meeting 6
To meditate 5 days a week, at 5 am	Yes	< ^1^	Yes	Yes	Yes
Call coworkers, physician, and psychologist		> ^2^			
Talk to husband about leisure together			No		
Read an item from the class				Yes	
Quit a few professional projects				Yes	
Final evaluation					
Question	Participant’s answer				
When I compare my expectations to my results, I think that:	I really saw the need to prioritize and I started the process of prioritizing between the lessons I took and in the areas where I can act. I learned a tool – [the ACT] Matrix – which will help me to walk through this process. I learned the importance of the group (any kind of group) in my personal project. Personal project does not mean isolation nor that will it happen if I isolate myself.				

^1^Meditated 4 days a week

^2^Also registered at a conference of interest

**Table 5:** Expectations, goals, and evaluations of participant 2

Expectations					
Question	Participant’s answer				
Why did I seek out this activity?	I have been in public service for 46 years; I never acted without being employed [by a company or government]; it’s time to think about what to do in the upcoming years. I do not want to waste what I learned. I know how to help people and I want to continue doing so.				
What do I expect to achieve by the end of the activity?	A plan for activities to be performed as a self-employed individual. Some possibilities for work. To learn more about life after retirement.				
Goals					
Activity	Life domain and meeting number				
	Health meeting 2	Career meeting 3	Leisure meeting 4	Career meeting 5	Couples meeting 6
Eat up to 3 slices of wholegrain bread per day	> ^1^	Yes	Yes	Yes	Yes
Drink at most 2 cups of coffee per day	Yes	Yes	< ^2^	Yes	Yes
List five options for future professional activities		> ^3^			
Do 2 leisure activities with wife			> ^4,5^		Yes
Structure and write down business plans				Yes	Yes
Pay attention to son’s bedtime					Yes
Final evaluation					
Question	Participant’s reply				
When I compare my expectations to my results, I think that:	The results exceeded my expectations greatly. I’ve learned with my group of colleagues how to think, plan, and share ideas about the future. I have to have challenging but attainable goals. The meetings helped to highlight the need to plan for the future based on social and psychological factors (the predictors) which interfere in people’s adjustment, in my adjustment, when I retire.				

^1^Changed his goal to up to 2 slices of bread per day

^2^Exceeded the goal limit

^3^Listed 10 options

^4^Happy hour, 2 movies at the theater, getting groceries, preparing lunch, riding a bicycle

^5^Reported that other changes started after the beginning of the intervention: eliminated after-work snack; reduced eating at night; lost 4 kg; at work, acted with less criticism and more calmly

**Table 6:** Expectations, goals, and evaluations of participant 3

Expectations				
Question	Participant’s answer			
Why did I seek out this activity?	I feel prepared for retirement, but I know it’s a new phase that will certainly bring some difficulty in adaptation. Exchanging ideas with other people who are going through the same process can help me see other aspects I have not considered.			
What do I expect to achieve by the end of the activity?	To be prepared to face a new challenge regarding the things which still worry me and to have contributed in some way to the other participants.			
Goals				
Activity	Life domain and meeting number			
	Health meeting 2	Career meeting 3	Leisure meeting 4	Friendships meeting 5
Drink no more than 3 coffees per day	Yes	> ^1^	< ^2^	
Watch three videos about financial education		Yes		
Go out with friend			Yes	
Make a list of friends I would like to meet again				Yes
Invite Sonia to visit her granddaughter				Yes
Do small favors				x
Final evaluation				
Question	Participant’s answer			
When I compare my expectations to my results, I think that:	My goals were reached as I identified the areas to be worked. The developed techniques helped me identify my difficulties and develop strategies to deal with them. The acquaintanceship with people involved in the same project made me think of other possibilities I had not considered until then. In the same way, I think to have contributed, in some way, to the group’s growth.			

^1^Changed the goal to no more than 2 coffees per day

^2^Exceeded the goal limit

^3^Participant 3 missed meeting 6; thus, she had no new goals. However, she emailed her task record for the week before

Table 4 shows that, with the exception of daily meditation, all the goals of participant 1 involve behaviors absent from her routine. After meeting 3, she surpassed her professional goal: beyond calling two colleagues working on the same subject as him, he also signed up for a conference on the topic. Of the eight goals, the participant failed to achieve only one: talking to her husband about leisure. In meeting 6, emotional, she reported feeling unable to do anything regarding her husband. With the collaboration of the group, she kept working in the marital area by means of a goal she felt she could accomplish: reflecting on her relationship daily for 10 min. In comparing the expectations to the final evaluation, it can be concluded that by using the matrix and group work, the participant achieved her objective of improving organization and emotional management.

Table 5 reveals that participant 2 accomplished all his objectives. The only exception was exceeding his daily coffee intake limit, but that was a goal from the second meeting he kept throughout the intervention “in solidarity” with participant 3, who originally proposed it. In the life areas of health, profession, and marital relationship, he exceed his goals: he implemented four dietary changes, which resulted in the loss of 4 kg 1 month after the intervention started; listed accomplishing the professional possibilities twice; interacted more calmly with coworkers; and did four activities with his wife, though he had planned only one. When comparing expectations to the final assessment, he achieved his objectives: he discussed retired life, identified new work possibilities, and began developing a business plan. Beyond this, he strengthened his belief in the importance of retirement preparation and learned to plan more achievable goals.

Table 6 shows participant 3 as having accomplished seven goals. Working in the friendship life area, he proposed three goals and achieved two. Comparing expectations to final assessment, it can be concluded that the intervention effects met his demands: the participant identified life areas of interest and chose an activity to dedicate himself to in retirement, a suggestion from participant 2. Additionally, he applied himself to his social life as much with intervention colleagues as friends, the area of greatest interest.

## Discussion

The study described in this research was undertaken with the objective of examining the social validity of a retirement education intervention based on CBS. The intervention presented socially relevant goals by stimulating participants to choose personally interesting tasks and allowing the adaptation of the intervention itself in accordance with their preferences and values. The intervention goals took previous skills into account and promoted new ones relevant to performance in different contexts.

The ability to individualize the activity while collaborating with coworkers meant that the intervention was able to attend to the categories proposed by Lane and Beebe-Frankenberger (2004), encompassing the participants’ already existing skills; adapting the intervention to personal preferences, values, and goals; and promoting skills relevant in differing situations. From the point of view of CBS, this result is based on the principle that the unit of analysis is the act-in-context: the intervention attributes to the individual or group, who possesses knowledge of the respective current and historical contexts, the role of establishing which values and goals are relevant, as well as selecting values and goals as a function of the work to be done (Hayes, Strosahl, & Wilson, 2012).

The procedures were mostly well-received, with emphasis on the role of the facilitator. In the proportion of comments on the facilitator’s performance, almost 40% of the total is consistent with evidence from other intervention implementations (Berkel, Mauricio, Schoenfelder, & Sandler, 2011; Santos & Murta, 2015), which indicate the relevance of facilitator behavior on the intervention’s outcome. The literature shows that this is an important therapeutic factor (Budd & Hughes, 2009) and could enhance the quality of mental health services (Laska, Gurman, & Wampold, 2014). Content that promotes significant personal reflection and well-organized and diversified material have also been shown to be important.

The ACT Matrix was the heart of the intervention. Working the matrix was the main procedure; it shaped identification of goals and proposal of tasks. It deserves special attention because it was used in almost every meeting, and participants adhered with no reservations and worked collaboratively according to each step of the matrix. Its integration with the other procedures is compatible with the instrument’s foundations: it was based on the ACT intervention model, which promotes acting according to values, and on the philosophical premise of the human being as a unitary organism, a CBS principle that behaviors, emotions, and physical sensations are on the same level and influence each other—thus, intervening in one of these elements can have repercussions in the others.

As for the procedures in general, however, operational adjustments are still needed. Examples include improving recruitment, fitting the content to the workload, and using more dynamic intervention methods. It is also necessary to improve the instruction and adjust the content to the available time. In part, the instruction can be improved via dynamic methods, such as experiential exercises as well as the adjusting of the material, inserting summaries and page numbers. However, the quality of instruction also depends on the available time. For example, the lack of sufficient time did not permit complete exposition of the material and limited discussion opportunities among the participants.

The intervention advanced socially important effects by means of sequential behavioral changes in line with the life domain being worked and compatible with the participants’ expectations. The results demonstrate the viability of instigating new behaviors based on significant values through retirement education. In the three groups, the sharing of values, aversive internal events, and dysfunctional behaviors occurred in a natural way and increased over the course of the intervention. While establishing goals, the participants collaborated, offering and soliciting suggesting to their peers. In general, achieving personally meaningful results reinforces the potential of interventions for retirement education (Leandro-França et al., 2016). The participants had quite diverse goals and, at times, they addressed distinct areas of life in the same meeting, but the focus on values presented a common thread, promoting group work and permitting the development of distinct interests. However, the absence of a follow-up did not permit evaluation of the maintenance of the new behaviors.

In accordance with the strategy that guided the intervention development, the high acceptability of effects is based on two principles of functional contextualism: prediction and influence of behavior. Both are distinct objectives, so an effective intervention must meet both. The analysis should be deep enough to boost the intervention’s effectiveness, leading to the optimization of time and targeting the intervention’s activities.

The intervention evaluated in this study also presents two important limitations. The first is the independence of the evaluator: preventive programs whose evaluations are done by their own creators tend to report more favorable results (Eisner, 2009). Due to the study conditions, however, external evaluation was infeasible. The second important limitation is the absence of a follow-up evaluation to verify the maintenance of social validity of the effects. Although the effectiveness evaluation exceeded the goals of these pilot studies, preventive interventions should conduct at least one long-term follow-up to evaluate the maintenance of its results (Gottfredson et al., 2015).

Based on the conclusions from the analysis of the three groups, it is possible to outline some next steps for research on the development of interventions for retirement preparation. From a public health perspective, the participant selection step should be done in a systematic manner by the researchers, minimizing sample bias and boosting external validity of the data. To foster implementation quality, in turn permitting a reliable measurement of the effects, it is important to work in closed groups. Finally, evaluating moderators and mediators in terms of, for example psychological flexibility, in efficacy studies can clarify the contextual aspects that interfere with the intervention’s functionality.

Solid and socially relevant knowledge production can boost scientific and social development. CBS brings a strategy for organizing and making sense of available knowledge, which should improve the quality of newly produced knowledge. Social validity complements this process, bestowing more meaning on the research. For many immersed in the academic world, the discrepancy between the effort involved in the production of knowledge and its low social impact is frustrating. Evaluating social validity may reveal this lack of impact, presaging receptiveness to a new intervention. In principle, this would permit modifications which foster the dissemination of the intervention, completing the cycle of research into prevention and the promotion of mental health as well as promoting quality of life.

## Conclusion

This study makes two principle contributions. The first is the use of CBS as a guide for developing interventions (Hayes et al., 2013). The construction of the intervention starting from philosophical premises, theories, and methods compatible with CBS shows the integration of suppositions with application. Contextual functionalism principles inspired goal planning, instrument selection, and behavior change activities. The promotion of values-based behaviors, leading to new protective behaviors, shows the emphasis of the intervention on functional and manipulable processes. The simplicity of the intervention, based on a manageable applied instrument with individualized goals, should facilitate its implementation and dissemination. The construction of a preventive group intervention with flexible objectives is compatible with the practicality demanded by public health care. The weekly monitoring of goals revealed the elevated commitment of the participants, a sign of the intervention’s acceptability. Also, the promotion of new behaviors in different areas of life demonstrates that the intervention has cross-sectional reach. Finally, these exploratory studies reinforce the expectation that the strategic argument proposed by CBS is a call to promising action (Hayes, Strosahl, & Wilson, 2012).

Within the intervention development, the use of CBS to develop a retirement education intervention stands out for this intervention’s preventive nature. Looking at the 144 clinical studies based on ACT published between 1986 and 2015, only seven mentioned prevention of negative outcomes or promotion of mental health in their titles (Association for Contextual Behavioral Science, 2016). Furthermore, there are no records of clinical studies based on ACT nor of publications related to CBS that address the adjustment to retirement (Association for Contextual Behavioral Science, 2016). Beyond this, the measurement of results via a daily record of behavior and not by interviews or scales is unique in retirement preparation interventions (Leandro-França et al., 2016). Therefore, the science of prevention and the practice of preparation for retirement can also benefit from the structure and contextual thinking of CBS.

The second contribution is the evaluation of the intervention’s social validity. Although social validity might foster the adoption and dissemination of an intervention, this is uncommon in the context of retirement. None of the 11 retirement preparation studies reviewed by Leandro-França et al. (2016) addressed social validity. All in all, only five evaluated the perceived satisfaction and only three undertook a needs survey prior to the trial. On the other hand, the relevance of social validity is clear: the effectiveness of an intervention is not enough to assure its acceptance by users—equally effective interventions are not necessarily equally accepted (Carter, 2010). While social validity is not sufficient to guarantee results, it is an important prerequisite for intervention implementation and dissemination in the real world.

In conclusion, according to the participants’ evaluations, the intervention provided socially valid goals, socially acceptable procedures, and socially important effects. However, some improvements are still needed, such as the use of more dynamic methods, better formatted printed material, and increased fidelity between the implementation of the content and the prescribed activities. The positive results indicate that contextual behavioral science may bolster the development of interventions based on components with evidence for social validity. The re-evaluation of the intervention via a clinical trial study should offer more robust evidence for its effectiveness. It is hoped that by increasing the availability of theory-based interventions in this area, the present study will promote valid strategies to facilitate better adjustment to retirement.

## Data Availability

Data are available from the first author (email: leopfq@gmail.com).

## Abbreviations

ACT: Acceptance and commitment therapy
CBS: Contextual behavioral science
SGP: Survey of Guiding Principles
TIDieR: Template for Intervention Description and Replication Checklist and Guide
VLQ: Valued Living Questionnaire
